# On-chip Extraction of Intracellular Molecules in White Blood Cells from Whole Blood

**DOI:** 10.1038/srep15167

**Published:** 2015-10-14

**Authors:** Jongchan Choi, Ji-chul Hyun, Sung Yang

**Affiliations:** 1 School of Mechatronics, Gwangju Institute of Science and Technology (GIST), Gwangju, 500-712, Republic of Korea; 2 Department of Medical System Engineering, Gwangju Institute of Science and Technology (GIST), Gwangju, 500-712, Republic of Korea

## Abstract

The extraction of virological markers in white blood cells (WBCs) from whole blood—without reagents, electricity, or instruments—is the most important first step for diagnostic testing of infectious diseases in resource-limited settings. Here we develop an integrated microfluidic chip that continuously separates WBCs from whole blood and mechanically ruptures them to extract intracellular proteins and nucleic acids for diagnostic purposes. The integrated chip is assembled with a device that separates WBCs by using differences in blood cell size and a mechanical cell lysis chip with ultra-sharp nanoblade arrays. We demonstrate the performance of the integrated device by quantitatively analyzing the levels of extracted intracellular proteins and genomic DNAs. Our results show that compared with a conventional method, the device yields 120% higher level of total protein amount and similar levels of gDNA (90.3%). To demonstrate its clinical application to human immunodeficiency virus (HIV) diagnostics, the developed chip was used to process blood samples containing HIV-infected cells. Based on PCR results, we demonstrate that the chip can extract HIV proviral DNAs from infected cells with a population as low as 10^2^/μl. These findings suggest that the developed device has potential application in point-of-care testing for infectious diseases in developing countries.

Infectious diseases, such as those caused by Human immunodeficiency, Ebola, Hepatitis, Influenza, and Dengue viruses, have been a leading cause of more than 50% of deaths in developing countries over the past decade^1^,^2^,^3^. For instance, since the first reported case of acquired immune deficiency syndrome (AIDS) in 1981, human immunodeficiency virus (HIV) has caused more than 39 million deaths as of the end of 2013, and an estimated 35 million people were living with HIV across the globe^4^. In addition, an estimated 240,000 children were newly infected with HIV from mother-to-child transmission in low-and middle-income countries in 2013^4^.

Although most infectious diseases are currently curable with proper treatment, millions of lives are lost or adversely suffered because the medical infrastructure in developing countries is inadequate for early diagnostic tests and subsequent treatments^5^. To develop diagnostics that rapidly identify infectious agents to provide timely treatment, the World Health Organization (WHO) has established a set of criteria whose initial letters form the acronym “ASSURED”: (i) affordable, (ii) sensitive, (iii) specific, (iv) user-friendly, (v) rapid and robust, (vi) equipment-free, and (vii) deliverable to those who need them^6^. In response to these demands, various miniaturized diagnostic tools have recently been developed for on-site disease detection. These tools, which employ a variety of techniques including enzyme-linked immunosorbent assay, lateral flow assay, electrochemical assay, or polymerase chain reaction (PCR) amplification, rapidly and reliably diagnose infectious diseases by analyzing biomarkers in blood plasma^7^,^8^,^9^,^10^,^11^,^12^,^13^.

Although plasma-based assays are widely used to detect diseases in prescreening tests, these approaches are limited compared with virus-infected cell analysis in their ability to diagnose viral infections^14^,^15^. First, antibody-based assays are effective only 3–6 weeks after initial infection, which may lead to false-negative test results of early infection^15^,^16^. Second, serological assays alone cannot directly identify viral infections transmitted from the mother in newborn infants at an early stage because maternal antibodies are directly transferred and may persist for 12–18 months after birth^17^,^18^. Third, plasma-based approaches cannot diagnose latent or persistent infections in which viral DNA is not cleared but remains in infected cells because the plasma levels of viral agents remain undetectable^19^,^20^. In contrast, virus-infected cell analysis enables decisive diagnoses of early viral infections, mother-to-child transmission, and latent or persistent infections^17^,^18^,^19^,^20^.

For these reasons, the analysis of intracellular biomarkers such as viral antigens, DNAs, and RNAs from virus-infected blood cells has emerged as an approach to facilitate more accurate, early, and confirmatory diagnosis of infectious diseases^17^,^18^,^19^,^20^. The first essential step is to extract the biomarkers in WBCs by separating the WBCs from other blood components that may interfere with the accuracy and reliability in diagnostic result and then lysing them. However, this step remains achievable only in laboratory settings, because the general protocol relies on reagents (lysis buffer, density gradient media), equipment (centrifuge), and a trained expert; these requirements severely limit access to extraction of viral agents from WBCs in low-resource settings^21^,^22^. Therefore, a sample preparation method that is user-friendly, inexpensive, disposable, efficient, and reagent/equipment-free should be developed for use in developing countries.

Microfluidic technologies have recently emerged as powerful methods to prepare blood samples in an automated, compact, rapid, and efficient manner with a small amount of patient blood^23^,^24^,^25^,^26^,^27^,^28^,^29^,^30^. Various individual cell separation or lysis methods based on mechanical^25^, chemical^26^, electrical^27^, and thermal^28^ principles have been proposed. However, a complete sample preparation device that simultaneously carries out both target cell separation and lysis has rarely been reported^31^,^32^,^33^. WBCs are in common isolated by filtration^31^, cell crossover^32^, or bead affinity^33^, after which they are chemically lysed in a microfluidic device. In those studies, the reported extraction efficiencies of genomic DNAs in WBCs from whole-blood samples were 35.7, 33.33, and 33.26 ng/μl, respectively. Because these devices may require accurate control of the flow rate with an additional chemical solution, equipment, or extra power source, their use is limited in developing countries.

Here we develop an integrated microfluidic sample preparation chip to efficiently extract intracellular components from WBCs without using of reagents, labelers, or routine lab procedures. To separate WBCs with high efficiency, a deterministic lateral displacement (DLD)-based device is designed with the serpentine channel for complete WBC isolation and two outlet channels with different volumetric flow rates for WBC self-enrichment. To continuously rupture the separated WBCs, a mechanical lysis chip with ultra-sharp nanoblade arrays (NBAs) are developed. An integrated microfluidic chip is then assembled from these two units to achieve on-chip WBC separation and lysis that is based on a mechanical mechanism only, without the need for sample dilution or additional reagents. Successful integration is demonstrated, through which the DLD and lysis chip are combined while the performance of the entire chip is retained. The device performance for efficient extraction of intracellular components from WBCs is quantitatively analyzed and compared with that for a conventional method in terms of total protein amount as well as genomic DNA (gDNA) purity and amount. As a potential point-of-care testing (POCT) application, HIV-infected cells in the blood are processed by the developed chip and its ability to extract HIV proviral DNAs is tested. The device is expected to rapidly provide useful biomarkers present in WBCs to POC diagnostics for early and confirmatory detection of various diseases in resource-limited settings.

## Results

### Working Principle of the Integrated Sample Preparation Chip

**Figure 1:**
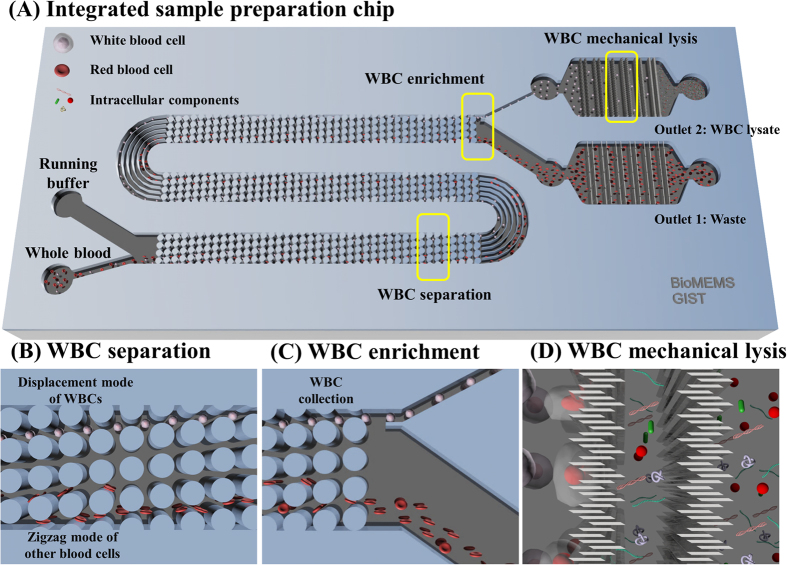
(**A**) A schematic illustration of the integrated sample preparation chip for continuous WBC separation and mechanical lysis. (**B**) Lateral displacement of WBCs is achieved by micropost arrays while RBCs flow in a zigzag mode. (**C**) Self-enrichment of WBCs is realized by controlling the width ratio between two outlets. (**D**) WBCs are simultaneously ruptured by mechanical nanoblade arrays with ultra-sharp edges to release the intracellular components in a continuous fashion.

We designed, fabricated, and developed an integrated sample preparation chip made of three polydimethylsiloxane (PDMS) layers and a mechanical cell lysis chip to extract intracellular components of WBCs from whole blood. Figure 1 shows a schematic diagram of the integrated microfluidic device and its working principle. The device consists of two inlet ports, DLD structures, mechanical lysis structures, and two outlet ports (Fig. 1A). Whole blood and phosphate-buffered saline (PBS) buffer are independently injected into the device from the two inlet ports. The blood sample becomes aligned along the left side wall of the inlet channel when the device is viewed from above with the outlet ports at the bottom. Large WBCs flow laterally and are continuously separated from the aligned main blood stream in the DLD device (Fig. 1B). Here, a serpentine channel with micropost arrays in the DLD device provides a long path for highly efficient WBC separation. The separated WBCs enter the narrower of two outlet channels; this gives rise to the self-enrichment effect, which increases the number of cells in the given volume (Fig. 1C). Lastly, the separated WBCs are mechanically ruptured by passing them through the NBAs with ultra-sharp tips to extract intracellular components (Fig. 1D). A dummy channel acts to balance the hydraulic resistance of the mechanical cell lysis channel to maintain the flow rate ratio between outlets 1 and 2. The developed chip continuously separates WBCs from whole blood and sequentially lyses them; thus, on-chip extraction of intracellular components in WBCs is realized without chemical reagents or a routine centrifugation process. A detailed description of the design and fabrication procedure for each device is provided in the supplementary material (Figs S1 and S2).

### Measurement of the Hydraulic Resistance in a Microfluidic Channel

In order to validate the channel design of each DLD outlet (for all three DLD types), and to confirm that the fluid flow between the dummy and lysis channels of the integrated device was well balanced, we measured the hydraulic resistances of each channel (Figs S3–5 and Table S1–5). It was found that the hydraulic resistances of both the DLD outlet channels and the dummy and lysis channels were in good agreement with those determined theoretically (specifically, the relative error in the comparison was less than 8.7%). We also found that the relative error in the comparison between the measured and calculated hydraulic resistance ratios of R_2_/R_1_ (e.g., outlet 2/outlet 1 of the DLD and lysis/dummy channel of the integrated chip) was less than 7.5%. Therefore, we concluded that the geometries of the DLD 3 outlets, along with the dummy and lysis channels of the integrated device, were set so as to balance the fluid flow.

### WBC Separation and Enrichment by the Separation Chip

The DLD chip was developed and its performance on WBC separation and enrichment was characterized. The designed critical diameter, D_c_, which is the criterion value for separating WBCs, was 4.6 μm (see SI Methods); the calculated D_c_ from the dimensions of the fabricated structure was 4.55 μm (n = 3). To test the present DLD device for separation of WBCs, a whole blood sample and PBS were injected at flow rates of 500 and 2,000 μl/h, respectively and the movement of each blood cell was investigated. WBCs exhibited a rolling motion without apparent cell deformation, and thereby laterally flowed to the main blood stream along micropost arrays (Fig. S8A). In contrast, a series of images taken by a high-speed camera confirmed that biconcave RBCs were vertically aligned, folded, or largely deformed near the micropost (Fig. S8A). The effective diameter of each cell (n = 10) from the micropost was determined using an image processing method (Fig. S8B). The obtained RBC effective diameter was 3.2 ± 0.7 μm, while that of the WBCs was found to be 8.4 ± 1.3 μm. Indeed, successful separation of WBCs was demonstrated (Fig. S8C). The collected sample from outlet 1 contained numerous RBCs without any WBCs (Fig. S8D), whereas that from outlet 2 included a number of isolated WBCs (Fig. S8E). Therefore, the device achieved highly efficient WBC separation by moving most WBCs to the collection outlet through the long separation path.

**Figure 2:**
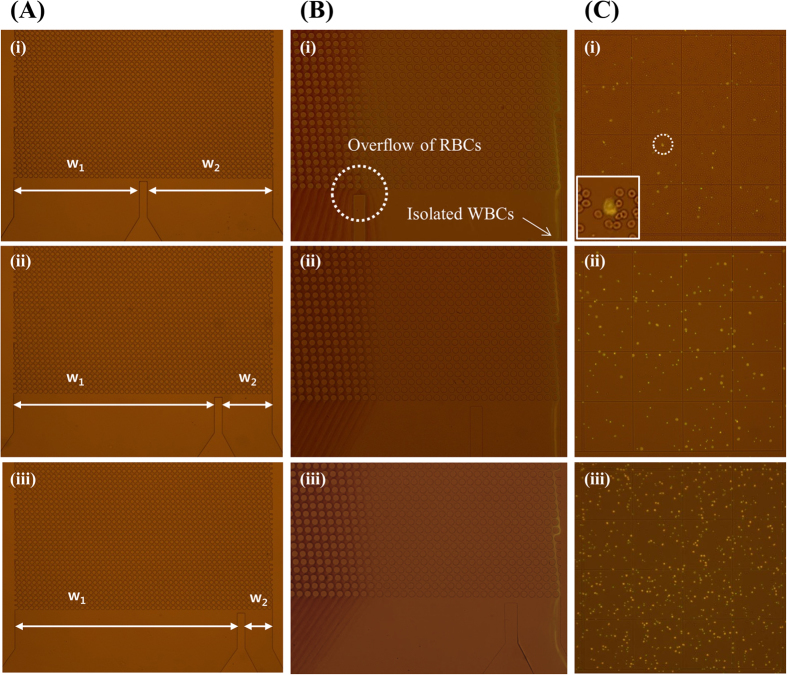
Effect of outlet width ratio of DLD device on WBC separation and enrichment. (**A**) Types 1, 2, and 3 represent the fabricated DLD devices with different outlet width ratios. (**B**) Most WBCs were successfully separated from whole blood to the running buffer flowing through the serpentine channel regardless of outlet width ratio. (**C**) Each isolated WBC sample was collected at outlet 2, and cells visualized under bright field and fluorescent illumination were counted using a hemocytometer. Higher self-enrichment was achieved for narrower collection channels. WBCs from a whole blood sample are selectively stained by acridine orange.

Figure 2 shows the WBC enrichment results by varying the outlet width ratio. Types 1, 2, and 3 represent the fabricated DLD devices with different outlet width ratios (w_1_:w_2_) of 1:1, 4:1, and 8:1, respectively (Fig. 2A). Most WBCs were thoroughly separated and collected at outlet 2 regardless of device type (Fig. 2B). However, some RBCs flow into outlet 2 when using a type 1 DLD device because the higher viscosity of the blood fluid results in nearly a 1:1 ratio of widths occupied by the blood and PBS in the DLD channel (Fig. S7). The largest population of WBCs collected from outlet 2 was observed when the type 3 DLD device was used (Fig. 2C). In case of the type 3 DLD device, the width (w_2_) of one of the outlet channels is narrower than another outlet channel (w_1_). For a given total flow rate, the volume of fluid collected from outlet 2 will be less as compared to outlet 1. Since most WBCs are collected from outlet 2, the number of cells in the given volume will be higher than type 1 and 2 devices.

**Figure 3:**
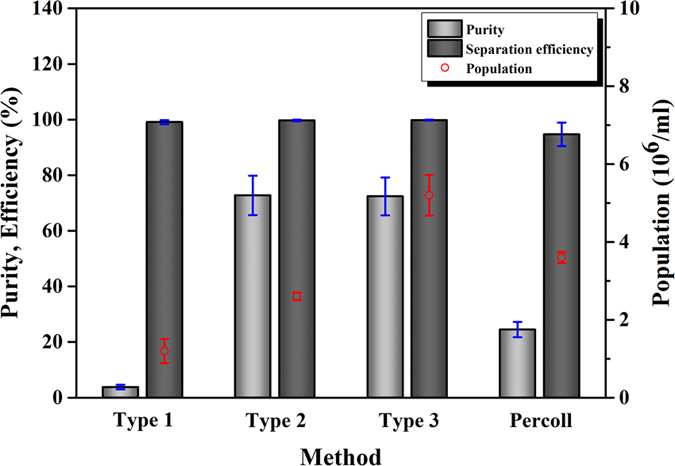
WBC separation results by DLD devices with various outlet width ratios and a commercially available Percoll solution. The purity, separation efficiency, and population of WBCs were determined by counting the target blood cells in a hemocytometer. Device type 3 (8:1 outlet width ratio) showed the best performance in terms of purity (72.4%), separation efficiency (99.8%), and population (5.2 × 10^6^/ml) of WBCs. The markers and error bars reflect the means and standard deviations of three measurements of the samples obtained from three devices. The difference between the devices has been depicted in Fig. 2.

Figure 3 shows graphical results of the purity, separation efficiency, and population of WBCs by using device types 1, 2, and 3, as well as a commercial Percoll gradient medium as a positive control. Percoll enables the complete separation of blood cells without cell damage^34^. Therefore, it was used as a positive control in a comparison of the separation efficiency of the conventional method and microfluidic device, regarding the separation of WBCs from whole blood. For the Percoll method, the purity, separation efficiency, and population of WBCs were 24.5%, 94.7%, and 3.6 × 10^6^/ml, respectively. The low sample purity was caused by some RBC contamination into the sample which contains WBCs during sample processing and manual sample collection.

The WBC purity calculated for device types 1, 2, and 3 devices were 3.8%, 72.8%, and 72.4%, respectively. As noted above, the low sample purity of device type 1 was due to the inflow of some RBCs into outlet 2. For types 2 and 3, the WBC purity was about 72%. We found that some RBCs did not show the certain deformation near the micropost and flowed laterally while keeping their effective size above 4.6 μm. A portion of these RBCs was partially included at outlet 2 and hence lowering the WBC purity. The WBC separation efficiency exceeded 99% (in many cases almost 100%) regardless of the outlet width ratio; that is, nearly all WBCs were recovered at outlet 2 through the DLD device. In addition, the WBC recoveries calculated for device types 1, 2, and 3 were 96.2, 93.6, and 95%, respectively, indicating some WBCs remained in the chip. Consequently, the developed device was able to separate nearly 99% of the WBCs with 72% sample purity.

We also investigated the effect of varying the outlet width ratio of the device on the self-enrichment of WBCs. For outlet width ratios of 1:1, 4:1, and 8:1, the population of WBCs increased as the outlet width ratio increased. The corresponding concentration factors (CFs) for the WBC separators were 0.26×, 0.59×, and 1.14× compared with the initial cell population before injection (4.55 × 10^6^/ml). In addition, CFs calculated by the collected volumes at outlet 2 were 0.27×, 0.63×, and 1.12× while that obtained from the theoretical calculation were 0.25×, 0.62×, and 1.17× respectively (Table S6). Device types 1 and 2 showed relatively low CF values, indicating that the separated WBCs were re-suspended in PBS much more than in the initial blood volume. In device type 3, the narrower channel induces a smaller volume to be collected at outlet 2, where re-suspension of the WBCs results in the enrichment of the cell population. Device type 3, which showed the best performance in terms of the purity, separation efficiency, and population of WBCs, was adopted as the cell separator for the integrated device for further studies.

We have also noticed that the shear rate applied in the microfluidic channel was in the hemolysis range^35^ (Fig. S7). We experimentally demonstrated that RBC hemolysis in the microfluidic channel does not affect the increasing protein concentration at outlet 2. It was found that apparent RBC hemolysis due to the high shear rate (over 1,000 s^−1^) was occurred in the main blood stream. However, it would not affect the increasing protein concentration because the released proteins or RBCs were not flowed into the outlet 2 (Figs S9 and 10).

### Intracellular Component Extraction by the Mechanical Lysis Chip

**Figure 4:**
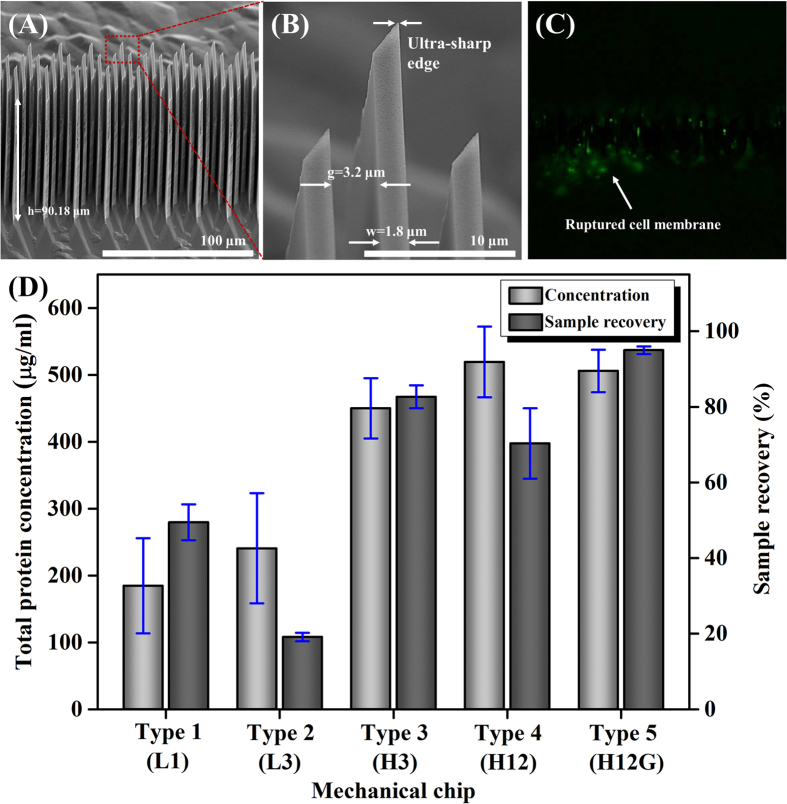
(**A**) Scanning electron microscope image of fabricated silicon nanoblade arrays (NBAs) with a high aspect ratio of 50:1. (**B**) Magnified image of ultra-sharp edge of NBAs whose width, gap, and tip were 1.8 μm, 3.2 μm, and several tens of nm, respectively. (**C**) A fluorescent image that experimentally demonstrates rupturing of WBCs by NBAs, using phalloidin eFluor® 520. Areas showing green emission represent ruptured and stained WBC membranes. (**D**) The mechanical lysis efficiency in terms of total protein concentration and sample recovery for different chip designs. The markers and error bars reflect the means and standard deviations of three measurements of the samples obtained from three devices.

The mechanical lysis chip embedded with ultra-sharp silicon NBAs was fabricated by a simple and cost-effective process of crystalline wet etching of (110) silicon, as we reported in a previous study^29^. Figure 4A shows a scanning electron microscope image of the fabricated silicon NBAs in the mechanical cell lysis chip. NBAs of 90.18 μm in depth were thoroughly constructed through anisotropic wet-chemical etching on a (110) silicon wafer. The width, gap, and length of the NBAs were 1.8, 3.2, and 31.5 μm, respectively (Fig. 4B). In addition, nanoscale ultra-sharp edges for mechanical cell disruption were fabricated by undercutting of the (110) silicon at convex corners.

To verify mechanical cell rupture by the NBAs, we used a fluorescent labeler to stain the filamentous actins (f-actins) beneath the cell membrane only when it is mechanically damaged or broken down. Figure 4C shows a fluorescent image of the ruptured WBCs. The WBCs mixed with 1× Phalloidin were flowed through the silicon channel with NBAs. We observed green light emission from the stained f-actins after 30 min at a flow rate of 500 μl/h. The observed fluorescence signal indicates that cell membranes were mechanically ruptured by the NBAs when the WBCs passed through the NBAs.

To improve the WBC lysis performance of the chip, we tested several devices designated as L1, L3, H3, H12, and H12G, where L and H denote low (36.01 μm) and high (90.18 μm) nanoblade structures, respectively, and the integer represents the number of NBAs in the series. The letter G indicates that the gap of the NBAs gradually changes from upstream to downstream along the length of the lysis channel. Detailed illustrations of the difference between the devices were depicted in Fig. S11. A 500-μl solution of WBCs in PBS isolated from whole blood by the discontinuous Percoll method was processed by each mechanical lysis chip. The collected sample volume and the intracellular total protein concentration in the cell lysate are presented in Fig. 4D.

Devices L1 and L3, which consist of low NBAs, showed the relatively low protein concentration and poor sample recovery. These devices did not fulfill the requirements for whole-sample processing, presumably because cellular debris was blocking the channel. Compared with L3, device H3 showed improved protein extraction and sample recovery. Device H12 showed some improvement in protein extraction; however, the recovered volume was slightly decreased than H3, presumably because of the high hydraulic resistance of the fluidic channel in H12 device. To increase sample recovery while maintaining the lysis efficiency, we tested device H12G, in which the gap between NBAs gradually varied from 13.2 to 3.2 μm in 2-μm decrements in series. Large gap (upstream) between nanoblade structures is adequate for rupturing the cell membrane, and narrow gap (downstream) is intended for subsequently lysing the nucleus. The results indicate that the efficiency of device H12G was superior to that of the other device, in terms of sample recovery and protein extraction.

### Confirmation of Continuous WBC Separation and Mechanical Lysis by the Integrated Chip

When attempting on-chip integration of the WBC separator and lysis chip, the maintaining the entry of separated WBCs to the mechanical lysis chip as well as the performance levels of WBC separation and lysis is important. We experimentally tested the integrated chip for continuous separation of WBCs from whole blood with a PBS buffer and their mechanical lysis (Fig. S12). These results suggest that as intended, WBCs were separated and also mechanically ruptured by being passed through the NBAs in the integrated microfluidic chip.

To confirm that the integration chip is elaborated for continuous WBC separation and lysis, changes in the trajectories of the WBCs and the main blood stream were examined with respect to processing time (Fig. S13). Initially, the relative measurement errors for the fluid widths of the main blood stream and WBC trajectory before and after integration were only 2.65% and 2.96%, respectively. This observation implies that the dummy channel in the integrated chip was effectively designed to balance the hydraulic resistance of the cell lysis channel. During the processing of 500 μL of whole blood, the width of the main blood stream narrowed by about 60.1 μm and WBC trajectory was also shifted by a distance of 40.28 μm. These changes are presumably caused by the stacking of cell debris in NBAs but have a negligible impact on device performance because the channel width of outlet 2 for WBC collection is 220 μm, which is sufficient to recover all WBCs.

### Quantitative Measurement of Cell Lysate by the Integrated Chip

The cell lysate prepared by the integrated chip was quantitatively analyzed by measuring the total concentration of intracellular protein and gDNA by using a bicinchoninic acid (BCA) protein assay kit and a UV-Vis spectrophotometer, respectively (n = 3 chips). The performance of the integrated chip was compared with the results of a chemical lysis buffer and mechanical lysis chip. Here, the measured concentration was then presented as the total amount by considering the recovered sample volume.

**Figure 5:**
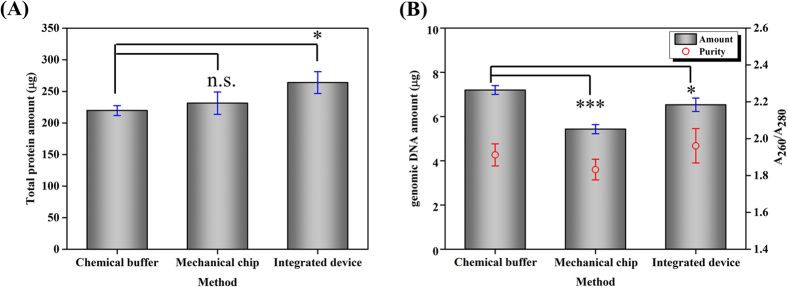
Quantitative analysis of (A) total protein amount and (B) purified gDNA amount as well as purity from cell lysates prepared by a commercially available cell lysis buffer, mechanical lysis, and an integrated microfluidic device. The total protein amount determined by chemical lysis was 219.6 ± 7.7 μg. The amount given by mechanical lysis was comparable, being 1.05 times that given by chemical lysis (n.s. *P* ≥ 0.05). In contrast, the amount given by the integrated device was 1.2 times that given by chemical lysis (*P < 0.05). The gDNA purity was between 1.79 and 2.06 for all samples, and the levels of gDNA concentration given by mechanical lysis and the integration device were 75% (***P < 0.001) and 90.3% (*P < 0.05), respectively, relative to the level given by the conventional method. The markers and error bars reflect the means and standard deviations of three measurements of the samples obtained from three devices.

The total protein concentration of the sample was determined by direct interpolation of a linear standard curve (y = 0.0002x + 0.0648, where R^2^ = 0.9923, y is the absorbance, and x is the sample concentration) relating the measured optical density with the known diluted albumin standards in the BCA protein assay kit. The total protein amount determined for chemical lysis was 219.6 ± 7.7 μg, whereas that for mechanical lysis and that for the integrated device were 1.05 times (231.3 ± 17.6 μg) and 1.2 times (263.9 ± 17.2 μg) greater, respectively (Fig. 5A). Mechanical lysis alone showed performance comparable to that of chemical lysis (n.s. *P* ≥ 0.05). This result suggests that intracellular components were thoroughly extracted by the breakdown of the cell membrane by the ultra-sharp NBAs. Moreover, the protein amount achieved with the developed sample preparation chip was significantly higher (*P < 0.05), presumably because self-enrichment of WBCs improved the protein extraction efficiency.

To evaluate the efficiency of nuclear lysis, gDNAs in the cell lysates were purified and the absorbances at 260 and 280 nm (A_260_ and A_280_, respectively) were measured to determine the DNA purity and amount (Fig. S14). The DNA purity, which is the ratio of A_260_ to A_280_, was between 1.79 and 2.06 for all samples (Fig. 5B). Hence, the purified sample contains pure gDNAs without proteins. The gDNA amount calculated from the measured A_260_ value was 7.2 ± 0.2 mg according to the conventional method; the amounts from mechanical lysis and the integrated device were 5.4 ± 0.2 and 6.5 ± 0.3 mg, respectively. The efficiency of gDNA extraction by mechanical lysis was nearly 75% (***P < 0.001) relative to conventional chemical method; that is, the mechanical lysis device was not sufficient for lysing all cell nuclei because the gap between nanoblade structures was limited to 3.2 μm. Nevertheless, the integrated chip showed a gDNA extraction efficiency of 90.3% compared with the conventional method (*P < 0.05). This suggests that WBC self-enrichment compensates for the low gDNA extraction performance of the mechanical lysis and improves the gDNA extraction efficiency in the integrated device. To check the integrity of the extracted gDNA in WBCs, gel electrophoresis was conducted and gDNA bands were found above the 10,200 bp of the DNA ladder marker, where gDNA is generally placed (Fig. S15). Given that no band appeared at any other position, we conclude that the gDNA prepared by the three methods was neither damaged nor fragmented.

**Figure 6:**
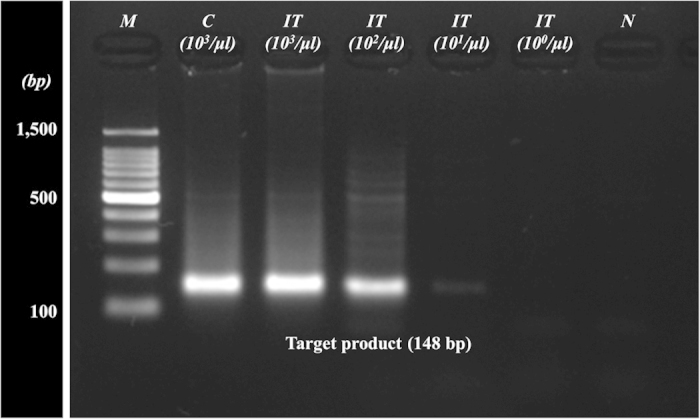
Agarose gel electrophoresis of the amplified HIV gag gene (148 bp) prepared by a general gDNA extraction and on-chip process. The integrated chip could successfully extract the HIV proviral DNA from infected cell populations as low as 10^2^/μl in the blood sample. Lanes: M, molecular weight standard; C, PCR product by chemical method; IT, PCR product by IT chip with respect to sample population; N, negative control.

To test the clinical applicability of the developed platform, we simply replaced the blood sample with one containing HIV-infected cells. The developed chip, when injected with a blood sample composed of RBCs, plasma, and HIV-infected cells, separated and lysed the HIV-infected cells. Furthermore, its ability to extract HIV proviral DNAs was validated by gel electrophoresis analysis of PCR products. The target PCR product (148 bp) of the HIV gag gene was comparably amplified in samples prepared by the conventional method (Lane C) and by the integrated chip (Lane IT) with a cell population of 10^3^/μl as shown in Fig. 6. It indicates that the extraction ability of gDNAs containing HIV proviral gene was comparable. Thereby, we diluted the samples by 10× fold and tested the detectable range of the cell population after processing the sample by the integrated chip. The target PCR band was clearly seen at the cell population of 10^2^/μl, but was weak at 10^1^/μl. From the results, the developed chip showed promise for clinical application because it could extract detectable HIV DNA at the cell population of 10^2^/μl.

## Discussion

In resource-limited settings, clinical sample preparation methods should be simple, inexpensive, efficient and capable of extracting clinical biomarkers for accurate diagnostic tests of infectious diseases. Beyond common serologic tests, analyzing intracellular biomarkers in virus-infected cells in whole blood enables conclusive, definitive, and confirmatory diagnosis of i) early viral infection; ii) mother-to-child transmission of infectious agents in newborn infants; and iii) latent or persistent infections.

To simply and efficiently extract intracellular proteins and nucleic acids in WBCs, a single-step sample preparation chip that performs continuous separation of WBCs from whole blood and their sequential lysis, was developed to replace existing sample preparation methods that are costly and laborious. The performance of the developed chip was demonstrated by quantitatively analyzing the intracellular protein concentration and gDNA levels from extracted samples.

The integrated device is more efficient than the conventional separation and lysis method in extracting intracellular proteins from WBCs (120% of the conventional method) and nearly as efficient as the conventional method (90.3%) in extracting nucleic acids (gDNAs). Thus, the developed chip guarantees levels of performance comparable to those of conventional methods in extracting intracellular proteins and gDNAs. Furthermore, we showed the feasibility of practically detecting HIV-proviral DNAs in HIV-infected lymphoblasts prepared by the developed device. The PCR product of the HIV-gag gene was detected by both the conventional method and developed device. The results revealed that the integrated device could extract HIV DNAs from infected cells with populations as low as 10^2^ cells/μl. Thus, the developed chip could facilitate on-site diagnosis of infectious diseases in combination with a compact POCT system such as ELISA, Western blotting, PCR, RT-PCR, or DNA microarray in resource-limited environments.

Although this sample preparation device relies solely on mechanical processes, it showed relatively low throughput sample processing. One strategy to improve the throughput to a flow rate of more than 500 μl/m is to lower the hydraulic resistance of the DLD channel by fabricating deep microposts. Then, a dual barrel syringe (4:1 volume ratio, MIXPAC™, Sulzer, Switzerland) designed to provide different input volume ratios could be directly connected to the chip for handheld sample preparation. The extracted gDNA also requires an additional purification process before proceeding to subsequent applications. Depending on the purpose of the assay, a nucleic acid purification process will be added to the chip by combining selective precipitation, silica-based materials, or magnetic beads to purify the nucleic acids from the WBC lysate.

Overall, the developed microfluidic device could achieve both separation of WBCs from whole blood and their sequential lysis in the absence of additional reagents and complicated processing steps. Its performance of extracting intracellular components from WBCs was also comparable to that of conventional methods. We also showed that proviral DNA in HIV-infected cells could be extracted from the blood sample, demonstrating the feasibility of the device for POCT application. Because the developed device provides the advantages of simultaneous WBC separation and lysis, as well as ease of use without any other chemicals or energy sources, except mechanical force, it promises to be an alternative to the conventional method for practical sample preparation in resource-limited settings.

## Methods

### Blood Sample Preparation

Whole fresh blood was supplied by a Biobank (Paik Hospital, Busan, South Korea). The whole-blood samples were used without pre-dilution in the experiment. The HIV-positive 8E5 cell line (CRL-8993™) was purchased from ATCC. The concentrated RBCs and blood plasma were obtained from a blood bank (Gwangju, South Korea) approved by the Korean Red Cross. The HIV blood sample was first prepared using concentrated RBCs and plasma at a hematocrit level of 43%. Then, the HIV-infected cells were spiked into the prepared sample at a final population of 10^6^/ml to 10^3^/ml. All handling of blood samples with HIV-positive cells was carried out in a biological safety cabinet in the biosafety level 2 laboratory. To visually distinguish the WBCs, we used acridine orange (A6014, Sigma-Aldrich, USA), a nucleic acid-specific fluorescence dye. An improved Neubauer hemocytometer (DHC-N01, INCYTO, Korea) was used to count cells.

The study was approved by the institutional review board (IRB) of Gwangju Institute of Science and Technology (GIST; 20140410-HR-11-02-02). All experimental protocols were approved by the IRBs of Buasn Paik Hospital of Inje University, Korean Red Cross, and GIST. The experiments were performed in accordance with the regulations and guidelines established by these committees. Signed informed consent was obtained from all participants and their anonymity was warranted.

### WBC Separation

A commercially available Percoll solution (Sigma-Aldrich, USA) was used to separate blood cells as a positive control. The difference in the density of cells is the major parameter for cell separation using Percoll solution. Peripheral blood mononuclear cells (PBMCs) and granulocytes were simultaneously isolated by centrifugation with 72% Percoll solution (Percoll, 1.8 ml; 10× PBS, 0.2 mL; 1× PBS, 0.5 ml) and 62% Percoll solution (Percoll, 1.575 mL; 10× PBS, 0.175 mL; 1× PBS, 0.75 mL) at 1,000 RPM for 30 min^34^. RBCs, PBMCs, and granulocytes were separately washed three times in PBS, re-suspended in PBS, and then counted. The total time required for WBC separation was approximately 1 h. A mixture of total WBCs with PBMCs and granulocytes was stored at 4 °C for further cell lysis experiments.

To separate WBCs using a DLD device, the microchannel was initially filled and incubated with 2% bovine serum albumin (BSA) for 1 h to minimize nonspecific binding of blood components. After the channel was carefully washed with 1× PBS, a whole blood sample and running buffer (i.e., PBS), each prepared in a syringe, were injected into the device through two inlet ports at flow rates of 500 and 2,000 μL/h, respectively by a syringe pump (NEMESYS, Germany). After discarding the remaining PBS in the channel, the samples obtained at an outlet port 1 or 2 were separately collected in a microcentrifuge tube through polyethylene tubing for 1 h. WBC separation in the DLD device was observed by capturing fluorescent images, using an inverted microscope (IX71, Olympus, Japan) with a CCD camera (DP 73, Olympus, Japan). In addition, high-resolution images of fine WBC or RBC motion were acquired by a high-speed digital camera (MotionPro X3, Redlake, USA). Each 10 μl of the collected samples from three different devices were examined by a hemocytomer for calculating the number of blood cells. Subsequently, the purity, separation efficiency, and population of WBCs were calculated for each sample.


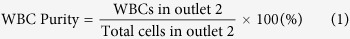



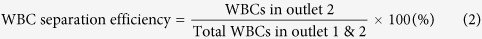



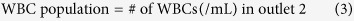



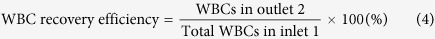


### WBC Lysis

For both chemical and mechanical lysis tests, WBCs were isolated from whole blood by the discontinuous Percoll method and divided into 500-μL aliquots of PBS solution. To prepare cell lysate from the integrated device, whole blood was directly used with a running buffer without any preprocessing. The cell lysis procedure requires approximately 1 h in total for the three methods.

For chemical lysis method, Cell lytic M (Sigma-Aldrich, USA), which is suitable for the protein extraction of mammalian cells, was used as the chemical reagent^36^,^37^. WBCs suspended in PBS were centrifuged at 1,000 g for 10 min; the PBS buffer was then totally removed, leaving a WBC pellet in the tube. A 500-μL solution of cell lysis buffer was added to the WBC pellet to lyse the WBCs in ice for 30 min, during which time the mixture was vortexed every 10 mins. The cell lysate in the tube was centrifuged at 1,000 g for 10 min and the supernatant was collected to obtain pure intracellular components.

For mechanical cell lysis, a syringe with a 500-μL solution of WBCs was connected to the mechanical lysis chip through polyethylene tubing. The solution was injected into the device by a syringe pump at a flow rate of 500 μL/h. The cell lysate was automatically recovered from the outlet reservoir. The purified intracellular components were collected from the supernatant obtained after centrifugation at 1,000 g for 10 min.

For the integrated device, the whole blood and PBS were injected at flow rates of 500 and 2,000 μL/h, respectively. WBC separation and mechanical lysis were simultaneously processed, and the resulting cell lysate was automatically recovered from outlet 2. The purified intracellular components were collected from the supernatant obtained after centrifugation at 1,000 g for 10 min.

### Quantitative Measurement of Cell Lysate Samples

For the total protein concentration in cell lysates, a bicinchoninic acid (BCA) assay kit (Thermo Scientific, USA) was used. Samples (10 μL) of diluted albumin standards ranging from 62.5 to 2,000 μg/mL, cell lysate, and PBS as a blank, were prepared in vials. Each sample was thoroughly mixed with 200 μL of working reagent—a mixture of 50 parts BCA Reagent A and 1 part BCA Reagent B. After transferring 200 μL of the mixture to each well of a 96-well EIA/RIA (enzyme immunoassay/radio immunoassay) plate, the plate was incubated at 37 °C for 30 min. The absorbance at 562 nm was measured on a plate reader (Biotek, USA) to acquire the optical absorbance of the sample cell lysates. A linear standard curve relating the measured optical density to the protein concentration was obtained from the known diluted albumin standards. The protein concentration of each unknown cell lysate was quantitatively determined from the standard calibration curve.

For the quantification of genomic DNA (gDNA) levels, the cell lysates were first processed with a gDNA purification kit (Solgent, Korea). To purify the gDNAs in the cell lysates, 200 μL of protein precipitation and 1 μL of RNase A were added to the lysates and the mixtures were incubated for 10 min. A pure supernatant was collected after centrifugation at 10,000 RPM for 1 min. An equal volume of isopropyl alcohol was added to the supernatant and the sample tube was inverted until the DNA precipitate was seen. After spinning down the sample at 10,000 RPM for 1 min, the supernatant was discarded and the remaining DNA pellet was washed with 300 μL of 80% ethanol twice. The purified DNA was then air-dried and re-suspended in 50 μL of DNA hybridization solution to give a final concentration of 10×. The absorbances of samples at 260 and 280 nm were measured by a UV-Vis spectrophotometer (Thermo Fisher, USA) to quantify gDNA purity and concentration after setting the calibration to zero with deionized water at the same wavelengths.

### Polymerase Chain Reaction (PCR) Process

PCR primers were designed on the basis of the full HIV-1 DNA sequence in the NCBI GenBank (NC_001802.1). The forward and reverse primers were selected by the Primer3 program (Whitehead Institute, Cambridge, MA, USA) to amplify a part of the gag gene (148 bp). The sequences of the forward and reverse primers were CACAGGACACAGCAATCAGG (5′ → 3′) and GGGTATCACTTCTGGGCTGA (5′ → 3′), respectively. Samples (1 μL) and the forward/reverse primers (each, 10 μM, 1 μL) were mixed with PCR premix (Taq polymerase: 1 U, each dNTP (dATP, dCTP, dGTP, dTTP): 250 μM, Tris-HCl (pH 9.0): 10 mM, KCl: 30 mM, and MgCl_2_: 1.5 mM, K-2012, Bioneer) and set to 20 μL with DI water. PCR was performed with a thermal cycle (30 cycles of 10 s at 95 °C, 30 s at 60 °C, and 10 s at 72 °C) by placing the reaction tube on the reaction chamber.

### Gel Electrophoresis and Imaging

To check the integrity of the purified gDNA, agarose gel (0.8%) was prepared in 1× TBE buffer. A gDNA solution of 4 μL was mixed with 1 μL of DNA staining buffer (Loading Star, DyneBio, Korea), together with a 1-kb DNA ladder marker (D-1040, Bioneer, Korea). Gel electrophoresis was run at 50 V for 1 h. The gel matrix was transferred to the ChemiDoc™ MP Imaging System (Bio-Rad, USA) for gDNA band imaging under UV illumination. For gel electrophoresis of the PCR product of HIV-DNA, agarose gel (1.2%) in 1× TBE buffer was first prepared. Each 2 μL of PCR product was mixed with 1 μL of DNA staining buffer and 3 μL of DI water, together with a 100 bp DNA Ladder (3407A, Takara). Gel electrophoresis was run at 100 V for 20 min and PCR bands in the gel matrix were visualized with the ChemiDoc™ MP Imaging System.

### Statistical analyses

To determine the WBC separation and lysis efficiency, the samples from each whole blood (500 μl) were collected from three different chips. The average and standard deviations were analyzed for each of these samples obtained from three chips. Data are expressed as mean ± standard deviation and two-tailed student’s t-test were used for all comparisons.

## Additional Information

**How to cite this article**: Choi, J. *et al*. On-chip Extraction of Intracellular Molecules in White Blood Cells from Whole Blood. *Sci. Rep*. **5**, 15167; doi: 10.1038/srep15167 (2015).

## Supplementary Material

Supplementary Information
