# Supplementary material for: On-chip Extraction of Intracellular Molecules in White Blood Cells from Whole Blood
Source: Sci Rep. 2015 Oct 14;5:15167. doi: 10.1038/srep15167 (PMC4604558; doi:10.1038/srep15167)
Supplement: Supplementary Information [file srep15167-s1.pdf]

On-chip Extraction of Intracellular Molecules in White Blood Cells from Whole Blood

Jongchan Choi[1]**, Ji-chul Hyun**[1]**, and Sung Yang**[1,2,*]

[1]School of Mechatronics, Gwangju Institute of Science and Technology (GIST), Gwangju, 500-712, Republic of Korea
[2]Department of Medical System Engineering, Gwangju Institute of Science and Technology (GIST), Gwangju, 500-712, Republic of Korea
*Correspondence and requests for materials should be addressed to S.Y. (email: syang@gist.ac.kr)

SI Methods

Device Design

DLD separation device

A deterministic lateral displacement (DLD) device based on differences in cell size is designed by setting two-dimensional micropost arrays inside the channel. The micropost diameter (D) is 30 µm, the gap (G) between microposts is 10 µm, and the center-to-center spacing (λ) is 40 µm. Each row is shifted perpendicularly to the direction of flow by Δλ (4 µm). Each row is thereby tilted 6° along the micropost arrays (Fig. S1A).

The designed critical diameter (D_c), which is the criterion for WBC separation, is 4.6 µm[1].

$D_c = 1.4 \times G \times N^{-0.48}$
$G = \lambda - D$
$N = \lambda/\Delta\lambda$

In addition, the post gaps (g_n) at the left and right side walls are differently modified to improve the WBC separation efficiency (n: 1, 2 … to 10, $\varepsilon = \Delta\lambda/\lambda$). The lateral displacement of cells is improved by letting more flux flow to the right side wall at every repeating separation junction within a row.
$g_{n,\,left}$ at the left side wall ranges from 3.16 to 10 µm in increasing order, and $g_{n,\,right}$ at the right side wall at the same row ranges from 13.78 to 10 µm in decreasing order[2].

$g_{n,\,left} = G\sqrt{n\varepsilon}$ for left side wall
$g_{n,\,right} = G\sqrt{2 - n\varepsilon}$ for right side wall

Enrichment design

The outlet channel of the DLD device is geometrically designed to produce a self-enrichment effect for isolated WBCs. The number of WBCs recovered at outlet 2 should at least be more than the initial population because the cell population is closely related to the concentration of extracted intracellular components. The concentration factor (CF) is defined as a volume ratio of the initial volume of blood ($V_{1,blood}$) to the output volume at outlet 2, where the separated WBCs are collected ($V_{2,DLD}$); $V_{2,DLD}$ is determined by the channel geometry of outlet 2. The output volume is a result of the flow rate at a given time, where the flow rate at outlet 2 is defined by the channel geometry, especially in terms of width ($w_{2,PBS}$). Based on the Poiseuille equation, CF could be expressed as the channel resistances of the of blood channel ($R_{1,blood}$) and outlet 2 channel ($R_{2,DLD}$). Given the theoretical expression, the WBC enrichment effect is estimated by varying the outlet width ratio (w_1:w_2) as 1:1, 4:1, and 8:1 (Fig. S1B). The corresponding DLD devices are designated as type 1, type 2, and type 3 separators.

$$\text{Concentration factor (CF)} = \frac{V_{1,blood}}{V_{2,DLD}} \approx \frac{\dot{Q}_{1,blood}}{\dot{Q}_{2,DLD}} \approx \frac{R_{2,DLD}}{R_{1,blood}} = \frac{\frac{12\mu_{PBS}L}{1-0.63\left(\frac{h}{w_{2,PBS}}\right)} * \frac{1}{h^3 w_{2,PBS}}}{\frac{12\mu_{blood}L}{1-0.63\left(\frac{h}{w_{1,blood}}\right)} * \frac{1}{h^3 w_{1,blood}}} = \frac{\mu_{PBS}(w_{1,blood}-0.63h)}{\mu_{Blood}(w_{2,PBS}-0.63h)}$$

Mechanical lysis chip

The nanoblade arrays (NBAs) of the mechanical cell lysis chip are designed with an initial mask pattern of a 300-µm-long (L_{mask}) and 3 µm narrow (G_{mask}) shaped rectangles which will be projected on a (110) silicon wafer from Cr mask (Fig. S1C). Each rectangle is fully arrayed parallel to the width of the mechanical lysis channel (4400 µm). The pattern gap between rectangles gradually varies from 12 to 2 µm in 2-µm decrements for every series of two rectangular pattern arrays aligned in the flow direction. Overall, a series of twelve rectangular pattern arrays is built along the length of the mechanical lysis chip to make a high-density NBAs. For converting the rectangles into NBAs, the alignment of the rectangular pattern with one of the vertical (111) planes on a (110) silicon wafer is a major factor. When the long side of the rectangular pattern is aligned in parallel with one of the vertical (111) planes, both long side walls are vertically etched down without undercutting. In contrast, both short sides of the rectangular pattern are significantly undercut, producing ultra-sharp edges in NBAs because the side wall is not aligned with the (111) crystalline plane[3].

Dummy channel

In the integrated chip, the two outlets are connected individually with a mechanical lysis channel and a dummy channel. While the separated WBCs flow through the mechanical lysis channel, the main blood sample (lacking WBCs) stably flows away through the dummy channel. The dummy channel is designed by considering the equivalent hydraulic resistance of the complex fluid network in the mechanical lysis channel. The height of the dummy channel is fabricated to be equal to that of the lysis channel. The geometric parameters of L_{dummy}, W_{dummy}, and G_{dummy} are determined from those of the fabricated NBAs (L_{blade}, W_{blade}, and G_{blade}), and the dummy channel is designed by varying G_{dummy} and W_{dummy} when L_{dummy} is equal to L_{blade}. By analogy between electronic and fluidic circuits, the width of the dummy channel is determined by the governing equation of Poiseuille's law for a channel with a particularly high aspect ratio where n is the number of NBA channels in parallel and α denotes the outlet resistance ratio[4] (Figs. S1D and S6C).

$$\Delta P = \dot{Q} \times R$$

$R = \frac{12\mu L}{w^3 h} = \mu \times R^*$ When the aspect ratio is particularly large in a microfluidic channel

$$R^*_{lysis} = \frac{1}{\frac{1}{R^*blade}+\frac{1}{R^*blade}+\cdots+\frac{1}{R^*blade}} = \frac{R^*blade}{n}$$

$$R^*_{dummy} = \alpha \times R^*_{lysis};\ \alpha = \frac{R^*_{outlet1}}{R^*_{outlet2}}$$

Fabrication of Unit Device and Assembly of the Integrated Device. The integrated microfluidic device presented here consisted of a four-layered structure made of 3 polydimethylsiloxane (PDMS) layers and a mechanical cell lysis chip (Fig. S2A). Briefly, the first PDMS layer consisted of a serpentine DLD channel with micropost arrays cast from a pre-patterned SU-8 mold (Fig. S2B). SU-8 2025, a negative-tone photoresist, was patterned on a silicon wafer by a standard photolithography process. Briefly, a solution of SU-8 was evenly spin-coated on the silicon wafer. After planarization, the SU-8 deposited on the silicon was soft-baked at 65 °C and 95 °C on a hot plate to evaporate the residual solvent and to fix the SU-8 on the silicon wafer. The baked SU-8 substrate was then exposed to UV irradiation at 15 $mJ/cm^2 \cdot s$ through a Cr mask, after which the SU-8 substrate was further baked at 65 °C and 95 °C on the hot plate to selectively crosslink the exposed SU-8 patterns. Finally, the unexposed area of the SU-8 was wet-chemically developed by a SU-8 developer and rinsed with IPA and DI water. A PDMS gel made of PDMS base and curing agent (Sylgard 184, Dow Corning Co., Midland, MI) mixed at a ratio of 10:1 was poured onto the prepared SU-8 mold and then cured in an oven at 80 °C for 1 h after removing bubbles in a vacuum chamber. The PDMS replica with DLD patterns was obtained by detaching it from the SU-8 mold. Inlet and outlet ports were then simultaneously formed by a micropunch.

The second PDMS layer with 4 via-holes (each 2 mm in diameter) was fabricated as an interconnection for the isolated WBCs and blood sample to flow into the mechanical lysis chip and dummy channel, respectively (Fig. S2C). A 1- to 2-mm-thick PDMS layer was evenly spread and cured on the substrate, and then peeled off from the substrate. Four via-holes (each 2 mm in diameter) were simply punched out on the PDMS membrane by using a micropunch.

A mechanical cell lysis device was simply fabricated by a crystalline wet etching process. 3-µm-wide and 300-µm-long patterns in a Cr mask were precisely aligned in parallel with the (111) vertical plane of a (110) Si wafer, after which the photoresist was patterned by a standard photolithography process. A silicon nitride layer pre-deposited on the wafer was selectively removed by using reactive ion etching (Fig. S2D i). The Si wafer was then dipped in a KOH solution (45 wt%, 80 °C) for crystalline wet etching (Fig. S2D ii). The ultra-sharp edge at the end of the microstructure was finally formed by undercutting at the convex corners during the wet etching process. The prepared Si wafer was electrostatically bonded to the glass wafer with inlet/outlet ports that were already defined by the through-hole process, using a sandblasting method (Fig. S2D iii, iv). Lastly, the bottom PDMS layer contained a dummy channel designed for stable fluid flow and an empty space for inserting the mechanical cell lysis chip (Fig. S2E). The SU-8 mold was prepared on a silicon chip with SU-8 2075, while a PDMS block (12 mm × 4 mm) was attached to where the mechanical lysis chip would be inserted to provide empty space. The PDMS layer was cured and peeled off from the master mold by using tweezers and a knife blade.

Finally, each unit element was then aligned using an alignment system consisting of a precise x, y, θ, and z stage, which was controlled by a hand-disc micrometer with 1-µm accuracy. The chips were irreversibly bonded to one another in sequential order after surface treatment by O_2 plasma to form the integrated chip.

SI Results

Measurement of the Hydraulic Resistance in a Microfluidic Channel. The pressure drop

along the microfluidic channel of each device was measured using a differential pressure sensor (24PCEFA6D, Honeywell, USA) as shown in Fig. S3. The pressure sensor was connected to the two ports of the microfluidic channel with a tygon tube filled with a PBS solution. A 10-V DC power supply was used to operate the pressure sensor. The pressure drops with respect to the injection flow rate were measured using a multimeter and were simultaneously recorded via LabVIEW. The inlet flow rate was precisely controlled from 0−8,000 µl/h using a syringe pump. The measured pressure drop was then calibrated to the actual pressure drop in accordance with the sensor sensitivity (70 mV/psi). The hydraulic resistance was then determined by dividing the measured pressure drop by the obtained flow rate. The theoretical hydraulic resistance was obtained by the equation denoted in Fig. S6. In order to calculate the effective viscosity (μ_{eff}) of the blood and PBS when co-infused in the channel (outlet 1 and the dummy channel), the equation that is most widely used in the literature for two-phase flow was used[5]. The viscosities of the blood and PBS were measured individually in advance using a conventional rheometer (HAAKE MARS, Thermo Electron GmbH, Germany), and were found to be 4.1 and 1.1 cP at high shear rate (at 1,000 s-1), respectively.

First, the transient pressure response and the relationship between the pressure drop and flow rate for individual channels were characterized. The results showed that the measured pressure drops were stably saturated within 1 min at the given flow rate in various microfluidic channels. Moreover, we found that the hydraulic resistances of the individual channels were quite consistent, regardless of the flow rates. A linear regression analysis indicated good linearity ($R^2 > 0.992$) for all cases, as shown in Fig. S4.

Secondly, the hydraulic resistances of the outlet channels of the DLD devices and the dummy and lysis channels of the integrated device were measured using two PBS buffers as a sample (inlet 1) and running buffer (inlet 2) (Fig. S5 (A)). The injection flow rates for inlets 1 and 2 were 500 and 2,000 µl/h, respectively. We compared the hydraulic resistances of the channels using the Poiseuille equation. For the single-phase flow case, the relative error in the experimentally measured hydraulic resistance and the theoretical estimation was less than 7 %. Therefore, we concluded that the geometries of the DLD 3 outlets, along with the dummy and lysis channels of the integrated device, were set so as to balance the fluid flow at certain ratios. Measured and calculated hydraulic resistances of each device were stated in Table S1 and 2.

Thirdly, whole blood and PBS were injected into the DLD and the integrated device as the sample (inlet 1) and running buffer (inlet 2). It was found that the hydraulic resistances of both the DLD outlet channels and the dummy and lysis channels were in good agreement with those determined theoretically (specifically, the relative error in the comparison was less than 8.7 %), as shown in Fig. S5 (B). We also found that the relative error in the comparison between the measured and calculated hydraulic resistance ratios of R_2/R_1 (e.g., outlet 2/outlet 1 of the DLD 3 and lysis/dummy of the integrated chip) was less than 7.5 %. Measured and calculated hydraulic resistances of each device were stated in Tables S3 and 4.

Estimation of Blood Viscosity in a Microfluidic Channel. Blood is a non-Newtonian fluid. Therefore, its viscosity tends to change with respect to the shear rate acting on the blood sample. When designing a device and conducting an experiment, the blood viscosity and consequent width occupied by the blood stream in the microchannel should be considered in advance[6]. By using a type 1 DLD device, which has a 1:1 outlet channel width ratio, the blood viscosity was experimentally observed with respect to the shear rate ranging from 15.7 to 1212.5 s^{-1} while the input flow rate ratio was fixed at 1:4 (Fig. S7). Details of the numerical values and constants used for calculating the shear rate and viscosity values are provided in Table. S7. In addition, we additionally measured the viscosity of the PBS using a conventional rheometer (HAAKE MARS, Thermo Electron GmbH, Germany), and was found to be 1.1 cP. We then calculated the blood viscosity in the microfluidic channel according to the shear rates. The injection flow rate of whole blood was set to 500 µL/h because the viscosity (4.2 cP) was relatively constant above 595.8 s^{-1} (shear rate of 250 µL/h).

Experimental approach,

$\dot{\gamma} = \frac{6\dot{Q}}{wh^2}$ for shear rate

$$\Delta P = \dot{Q}_{blood} R_{blood} = \dot{Q}_{PBS} R_{PBS}$$

$$\frac{12\mu_{blood}L}{1-0.63\left(\frac{h}{w_{blood}}\right)} * \frac{1}{h^3 w_{blood}} = \frac{\dot{Q}_{PBS}}{\dot{Q}_{blood}} * \frac{12\mu_{PBS}L}{1-0.63\left(\frac{h}{w_{PBS}}\right)} * \frac{1}{h^3 w_{PBS}}$$

$\mu_{blood} = \frac{\dot{Q}_{PBS}}{\dot{Q}_{blood}} * \mu_{PBS} \frac{1-0.63\left(\frac{h}{w_{blood}}\right)}{1-0.63\left(\frac{h}{w_{PBS}}\right)} * \frac{w_{blood}}{w_{PBS}}$ for viscosity

RBC Hemolysis in a Microfluidic Channel. We have noticed that the shear rate applied in the microfluidic channel was in the hemolysis range[7]. First, in order to evaluate the hemolysis of RBCs in phosphate-buffered saline (PBS), the plasma in whole blood was completely replaced with a PBS buffer. We measured the protein concentration of the PBS solution after 1 h with a bicinchoninic acid (BCA) assay. It was found that the hemolysis was negligible as shown in Fig. S9. In another set of experiments, we collected samples at outlets 1 and 2 using a DLD type-3 device. The protein concentration was then evaluated (Fig. S9). The result from outlet 1 showed apparent RBC hemolysis due to the high shear rate (over 1,000 s^{-1}). In contrast, there was no detectable protein concentration in the sample collected at outlet 2. This is because the majority of the RBCs did not flow into outlet 2. In addition, we conducted another experiment to confirm that some proteins caused by the hemolysis in the main blood stream do not flow through outlet 2. Specifically, we added a 1-μM fluorescein isothiocyanate (FITC)-dextran solution to the whole-blood sample and measured the fluorescent intensity in the inlet and outlet channels. The FITC-dextran diffusion was observed in the outlet at 220 μm (Fig. S10). However, it was found that the dye did not diffuse to the outlet 2, where the WBCs were collected and lysed in this study. Based on the above results, we concluded that RBC hemolysis did occur in the main blood sample, but it did not influence the results of the sample analysis conducted at outlet 2.

Confirmation of Continuous WBC Separation and Mechanical Lysis. Fig. S12 shows images depicting the separation of WBCs from whole blood and their mechanical lysis by the developed sample preparation chip. The acquired images show that most RBCs flow straight through outlet 1, passing through the dummy channel (Fig. S12A i). Meanwhile, concentrated WBCs stained with AO were observed at the mechanical lysis chip (Fig. S12A iii). The sample collected from outlet 1 contained mainly RBCs (1.2×10^9/ml) with some amount of WBCs (1×10^4/mL) (Fig. S12B i). The sample collected from outlet 2, where WBCs passed through the mechanical lysis chip, was clean, but showed some residue of cell components (Fig. S12B ii). These observations suggest that WBCs were well separated and injected into the mechanical lysis chip. In addition, it suggests that WBCs were also mechanically ruptured after passing through the NBAs from the integrated microfluidic chip.

Fig. S12C and D show changes in the trajectory of blood cells with respect to time for both separated WBCs and non-separated blood cells during processing of a blood sample. Initially, non-separated blood cells and separated WBCs flowed thoroughly as expected (Fig. S12C i, ii). After 60 min, the width of the main blood stream slightly decreased, becoming compressed toward the left sidewall; in addition, the distance of the WBC stream from the right side wall slightly increased near the collection channel because of an unwanted blocking effect of cellular debris among the NBAs (Fig. S12D i, ii).

Fig. S6 shows the changes in the fluid flow pattern for both separated WBCs and main blood stream during sample processing with respect to time in the integrated device. The initial widths occupied by the main blood stream in the DLD and integrated devices were 1132.9 and 1102.8 μm, respectively. The standard error of difference was only 2.65%. The initial distances of the WBC trajectory from the right side wall were 26.28 and 25.5 μm in the DLD and integrated devices, respectively. This observation implies that the dummy channel in the integrated chip was effectively designed and fabricated to balance for the hydraulic resistance of the cell lysis channel. During the processing of 500 μL of whole blood, the total width of the RBC fluid stream decreased by 60.1 μm as the interface of fluids between blood and PBS flow shifted toward the left side wall. The trajectory of the WBC also changed as its distance from the right wall increased by 53.28 μm (from 25.5 to 65.78 μm).

References

1 Davis, J. A. *et al.* Deterministic hydrodynamics: taking blood apart. *Proc. Natl. Acad. Sci.* **103**, 14779-14784, doi:10.1073/pnas.0605967103 (2006).

2 Inglis, D. W. Efficient microfluidic particle separation arrays. *Appl. Phys. Lett.* **94**, 013510, doi:10.1063/1.3068750 (2009).

3 Yun, S. S. *et al.* Handheld mechanical cell lysis chip with ultra-sharp silicon nano-blade arrays for rapid intracellular protein extraction. *Lab chip* **10**, 1442-1446, doi:10.1039/b925244d (2010).

4 McGrath, J., Jimenez, M. & Bridle, H. Deterministic lateral displacement for particle separation: a review. *Lab chip* **14**, 4139-4158, doi:10.1039/c4lc00939h (2014).

5 Abdelkader S., Mostafa F., Jacques P., Judith S. Oil-water two-phase flow in microchannels: Flow patterns and pressure drop measurements, *Can. J. Chem. Eng.* **86**, 978-988, doi: 10.1002/cjce.20108 (2008).

6 Kang, Y. J., Yoon, S. Y., Lee, K. H. & Yang, S. A highly accurate and consistent microfluidic viscometer for continuous blood viscosity measurement. *Artif. organs* **34**, 944-949, doi:10.1111/j.1525-

1594.2010.01078.x (2010).
7 Yasuda, T. et al. Influence of static pressure and shear rate on hemolysis of red blood cells. *ASAIO journal* **47**, 351-353 (2001).

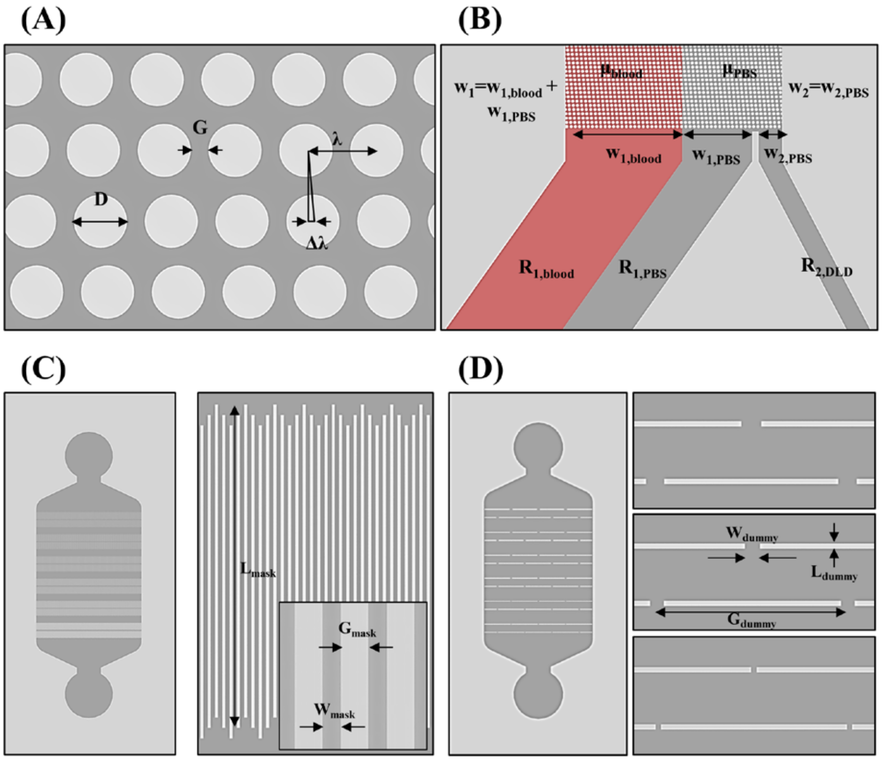

Fig. S1. Design of unit module for the integrated microfluidic device. (A) Design parameters of micropost array of DLD device (D, G, λ, and Δλ). (B) Design parameters of WBC self-enrichment. (C) Initial etch mask of mechanical lysis structure. (D) Dummy channel design parameters.

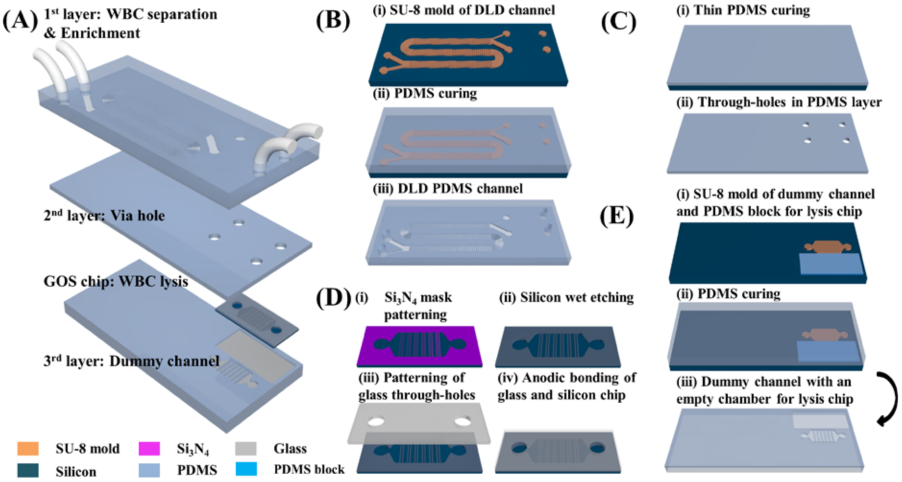

Fig. S2. Fabrication process of the integrated microfluidic device. (A) A bird's-eye view of the disassembled device. Fabrication procedures of the (B) 1st layer, (C) 2nd layer, (D) GOS chip, (E) and 3rd layer.

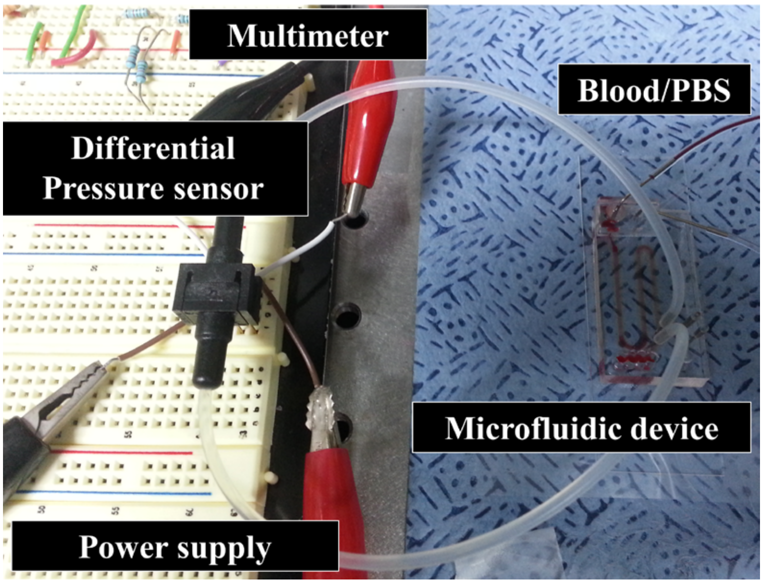

Fig S3. Experimental setup for measuring actual pressure drops and flow rates. DC power (10 V) was supplied to the differential pressure sensor, and the pressure drop along the microfluidic channel was recorded by a multimeter. The pressure data were collected and analyzed using the LabVIEW program.

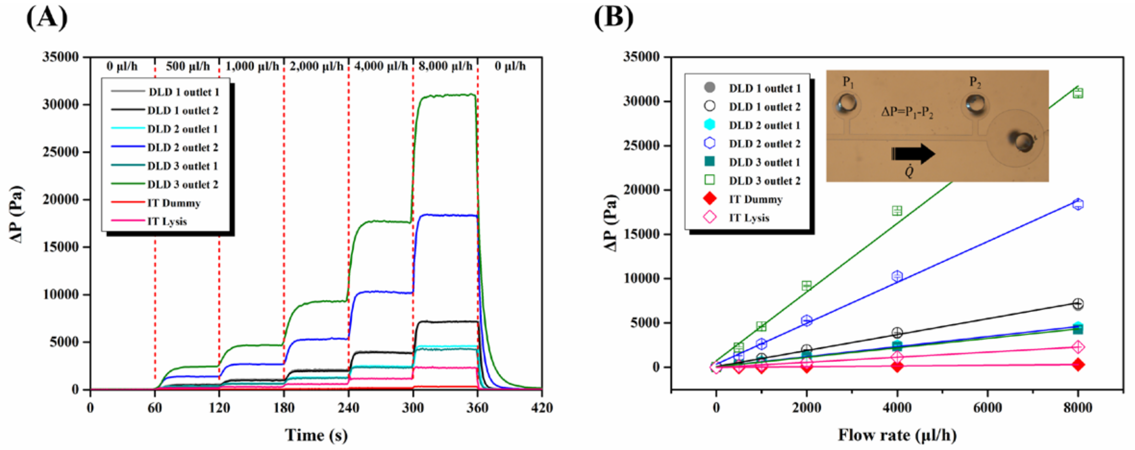

Fig. S4. (A) Transient pressure response of differential pressure sensor for increasing flow rates. The flow rates were controlled by a syringe pump from 0 to 8,000 µl/h every 1 min. The measurements were performed using a PBS in an individual channel, where the channel width was identical to the width of each device. (B) Relationship between measured pressure drop and injected flow rate for different channel widths. The slopes indicate the hydraulic resistances of each microfluidic channel when the sample fluid was PBS. From the linear regression analysis, the results show large linearity ($R^2 > 0.992$) for all cases. Three devices were used for the experiments.

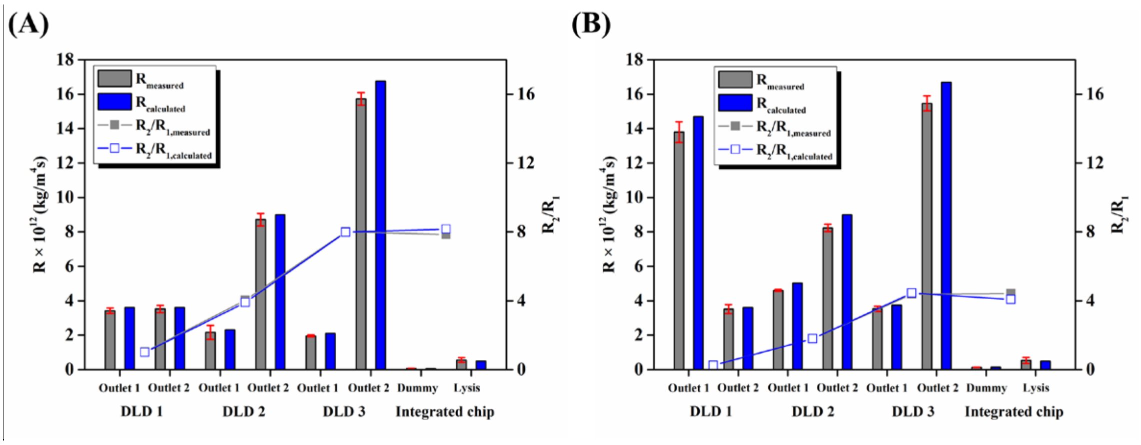

Fig. S5. Comparison of theoretical and experimental hydraulic resistances of each channel when (A) Two PBS buffers and (B) Whole blood and a PBS buffer were injected into the DLD and IT chip as the sample and running fluid, respectively. The left and right y-axes indicate the hydraulic resistance value of each channel and the hydraulic resistance ratio between the outlets 1 and 2, respectively.

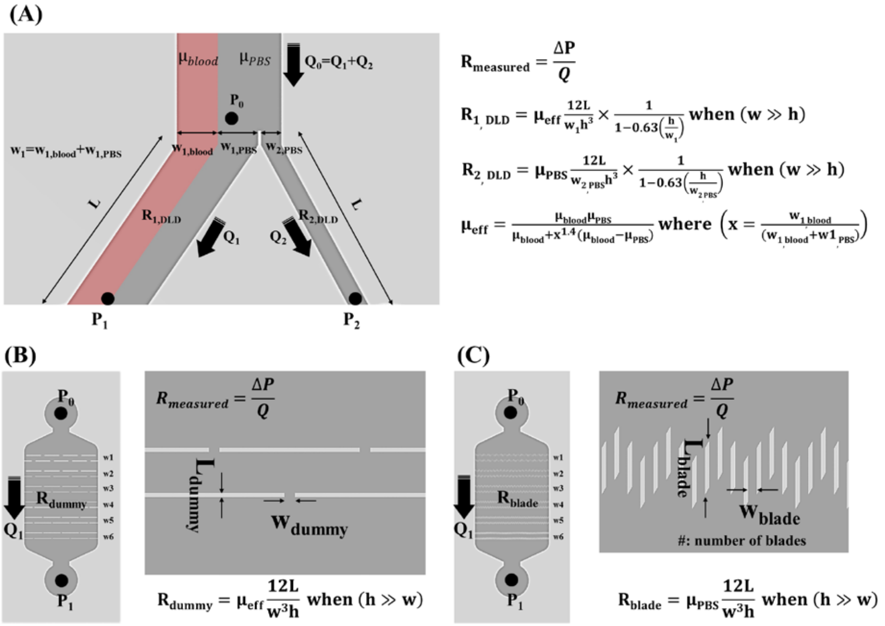

Fig. S6. Illustration of microfluidic network modeling of each device for theoretical calculation of the hydraulic resistances. (A) DLD channel, (B) dummy channel, and (C) mechanical lysis channel.

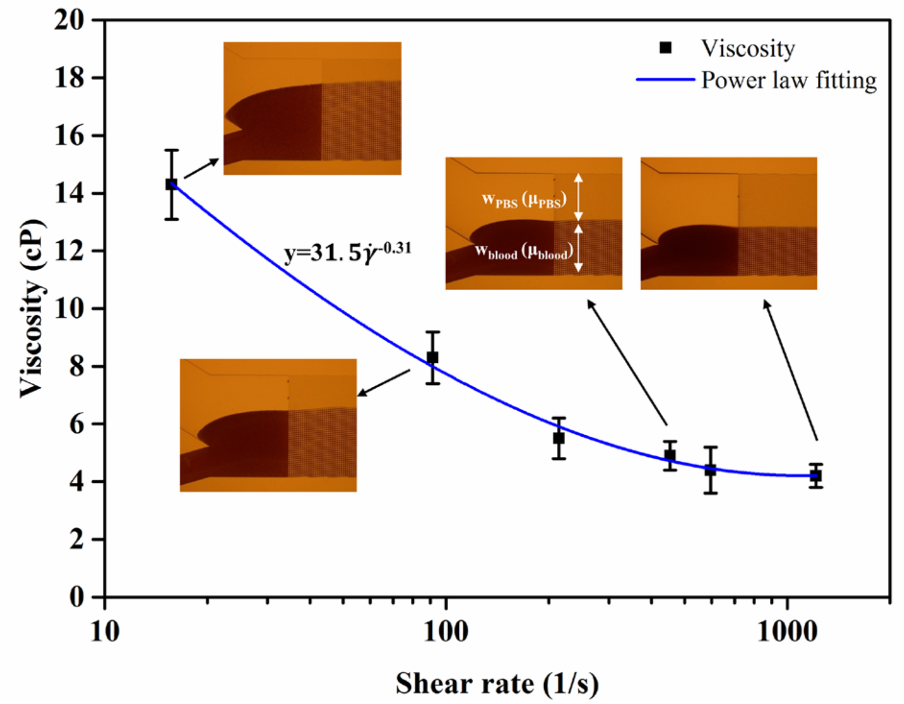

Fig. S7. The calculated blood viscosity with respect to shear rate. Blood becomes less viscous at high shear owing to cell deformability and disaggregation. The measured viscosity and width of the blood stream in the microfluidic channel become relatively constant at shear rates above 598.5 s^{-1}.

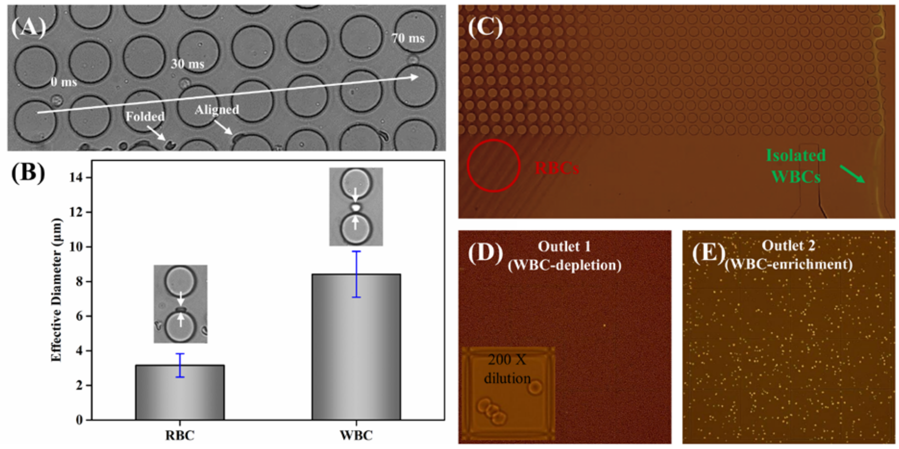

Fig. S8. Experimental results of WBC separation device. (A) The movements of RBCs and a WBC, as observed by a time-lapsed image captured by a high-speed camera, differ because the effective cell diameter varies depending on cell shape and deformability. WBC images captured at 0 and 70 ms are added to the image taken at 30 ms. (B) The effective diameters of both the RBCs and WBCs near the micropost were analyzed using the imageJ program, and were found to be 3.2 ± 0.7 and 8.4 ± 1.3 µm, respectively. (C) A fluorescent image of WBC separation is taken at the device outlet. (D) A hemocytometer image of the sample collected from outlet 1. The inset, which shows a 200× diluted sample, confirms that the majority of blood cells in outlet 1 are RBCs. (E) A hemocytometer image of the sample collected from outlet 2. WBCs from a whole blood sample are selectively stained with 1 µM acridine orange.

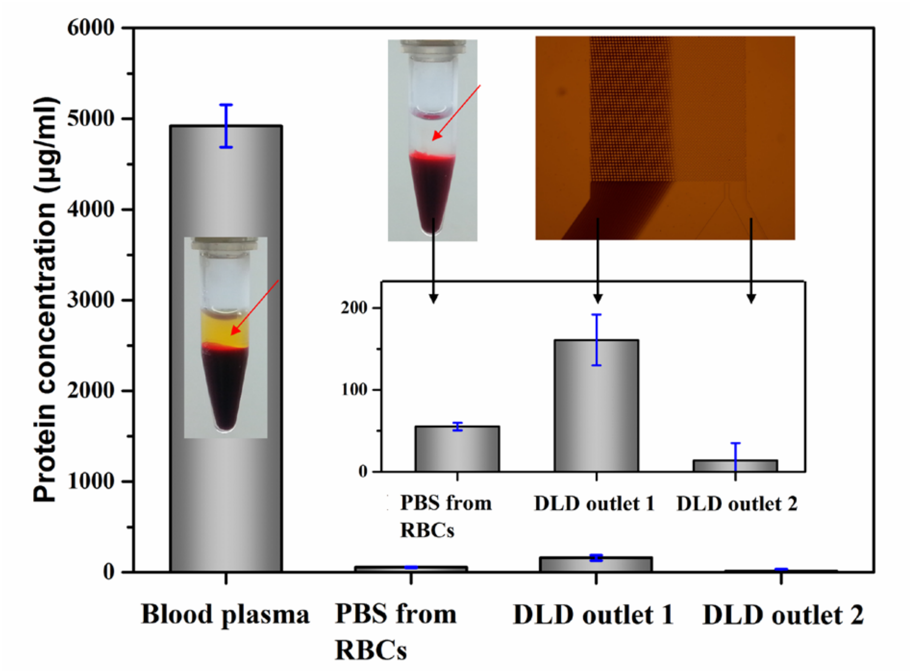

Fig. S9. The result of protein concentration measurement of the blood plasma, PBS from RBC suspension, and the samples from outlet 1 and outlet 2 in the DLD 3 device. No detectable protein concentration was found in the outlet 2.

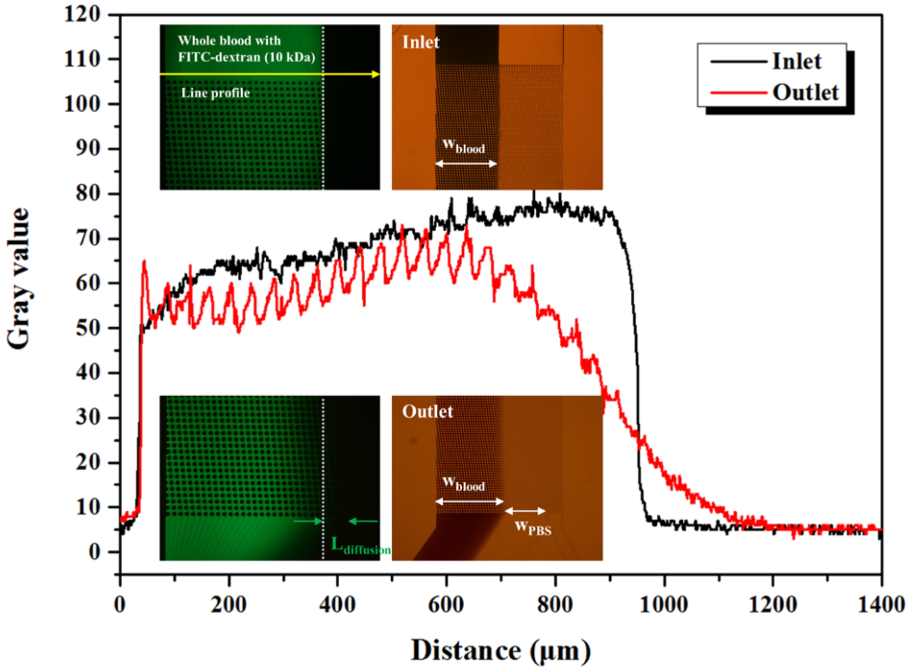

Fig. S10. FITC-dextran diffusion when diluted in whole blood. The upper inset images represent a whole-blood infusion containing FITC-dextran (10 kDa, 1 µM) and PBS at the device inlet. The line profile is plotted as a black line. The bottom fluorescent inset images indicate the diffusion of FITC-dextran from the main blood stream at the device outlet. The diffusion length of the FITC-dextran along the channel width was approximately 210 µm.

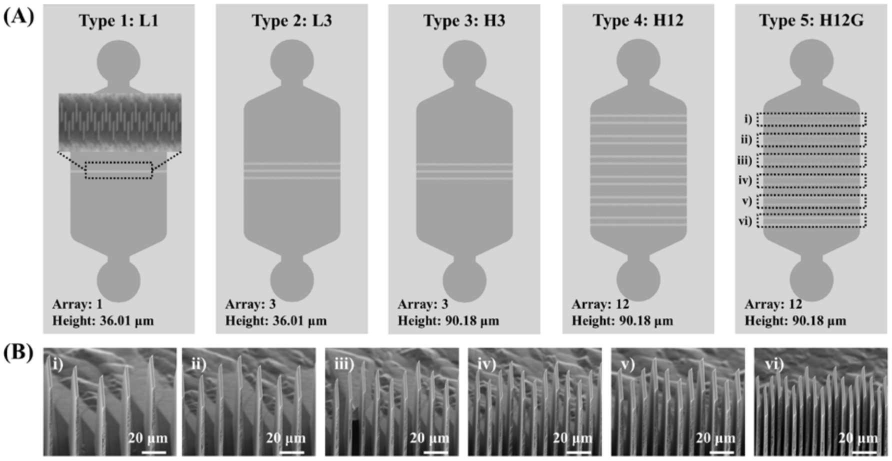

Fig. S11. Device configurations of various mechanical lysis chips used for the cell lysis test. (A) The scanning electron microscopy (SEM) images represent the blade array with different upstream-to–downstream gaps (from 13.2 to 3.2 μm with a 2-μm decrement) of the type 5, (B) H12G device.

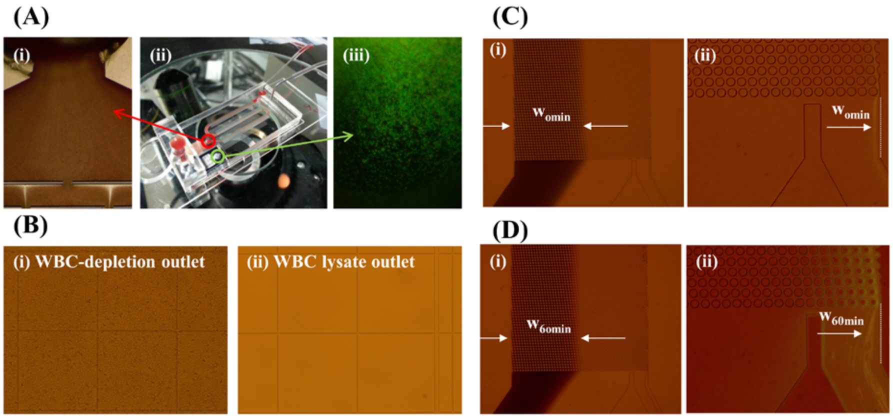

Fig. S12. Experimental demonstration to test the performance of the integrated sample preparation chip. (A-i) Flow of non-separated RBCs into the dummy channel. (A-ii) Separation of WBCs from whole blood and their lysis by the developed sample preparation chip, using a PBS buffer. (A-iii) Fluorescently stained WBCs were identified in the lysis device. (B) Hemocytometer images of the samples collected from each outlet. (B-i) Sample from outlet 1 was abundant in RBCs, but depleted of WBCs. (B-ii) Sample passed through the mechanical lysis device was clean, but contained some components resulting from WBC rupture. (C) Initial fluid stream of (C-i) RBCs and (C-ii) WBCs. (D-i) The width of the main blood stream slightly decreased after 60 min. (D-ii). A fluorescent image showing a shift in the WBC streamline away from the right sidewall after 60 min.

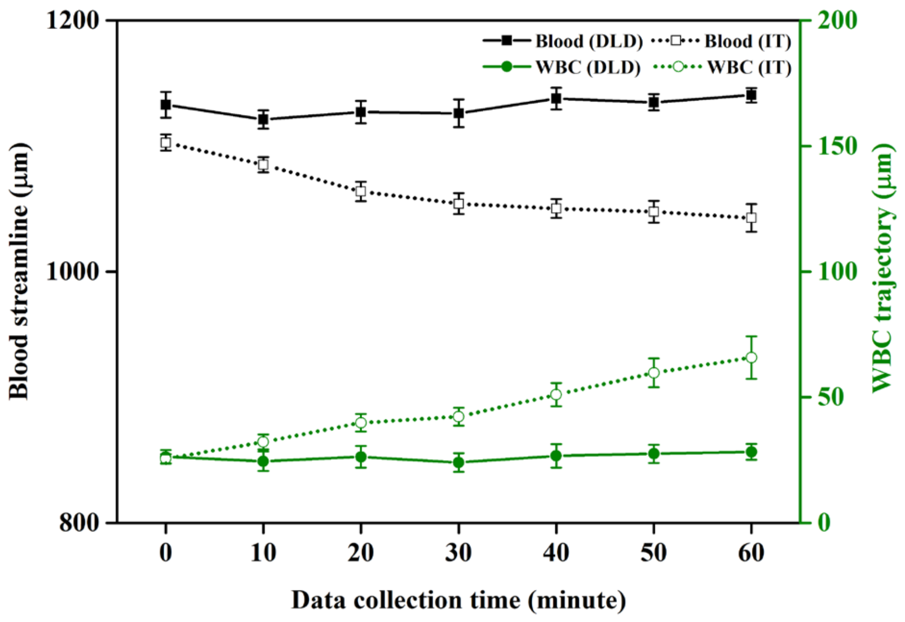

Fig. S13. Changes in the fluid flows of the main blood stream and WBCs in the DLD and integrated devices were observed to assess long-term device use with respect to data collection time.

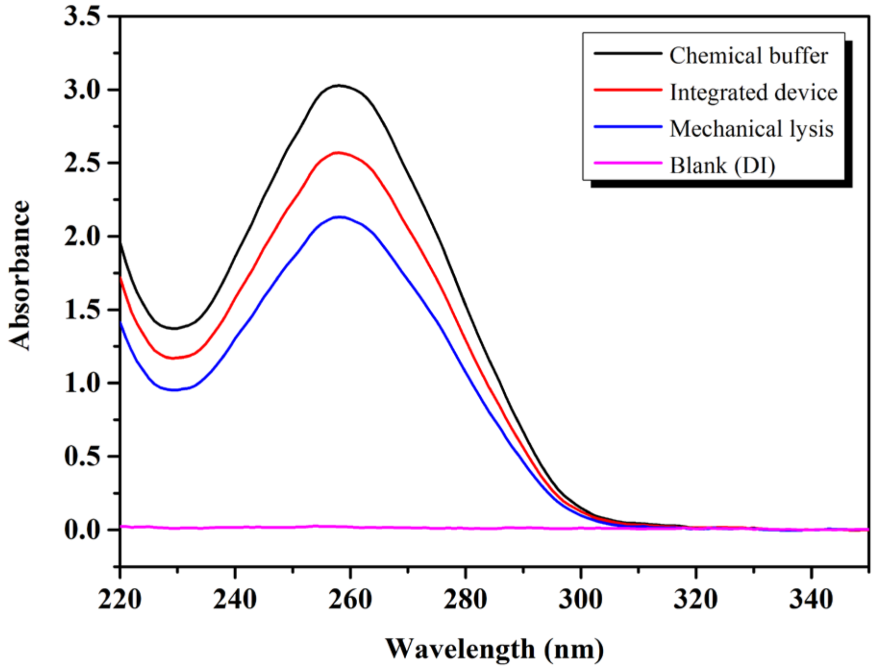

Fig. S14. Result of UV-spectrophotometry. The absorbances of gDNAs extracted by the three methods and purified by a conventional purification kit were measured with respect to wavelength.

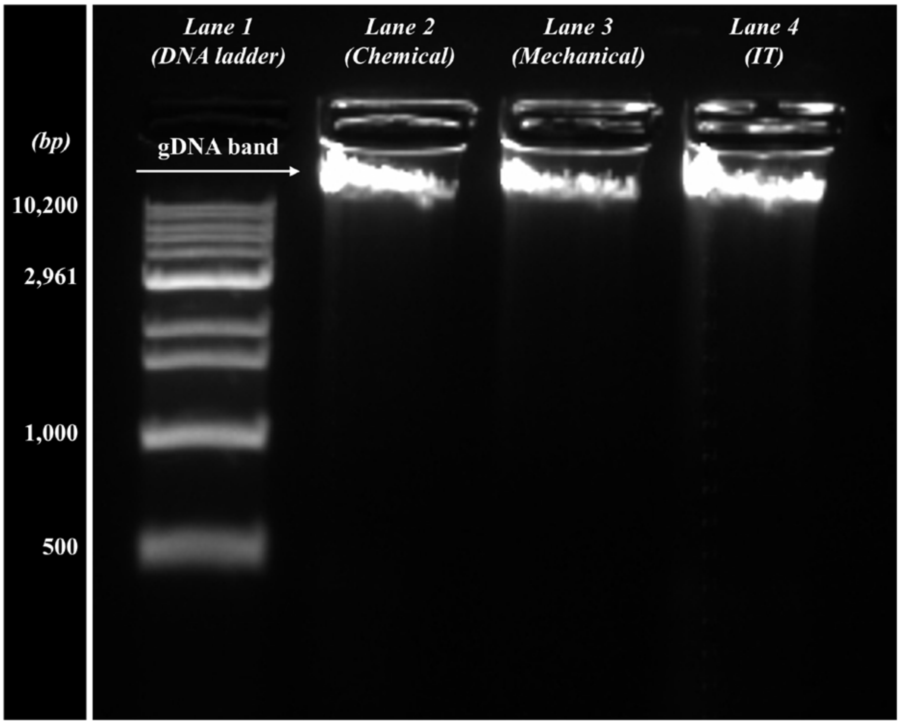

Fig. S15. Gel electrophoresis results to verify the integrity of gDNAs. Lane 1 represents the DNA ladder marker; lanes 2–4 show results for lysates prepared chemically, mechanically, and by the integrated device. Lane 5 represents the negative control. No additional band is apparent in lane 3 or 4, and a single band is seen in the same position for all prepared samples above 10,200 bp, where the gDNA band generally located.

Table S1. Measured hydraulic resistances of each device when two PBS buffers were used as a sample and running buffer. ΔP, Q, and R represent the pressure drop, flow rate, and hydraulic resistance.

Device	DLD 1		DLD 2		DLD 3		IT	
	Outlet 1	Outlet 2	Outlet 1	Outlet 2	Outlet 1	Outlet 2	Dummy	Lysis
ΔP (Pa)	1208.4	1255.4	1178.4	1219.5	1260.9	1327.6	48.25	42.1
Q (μl/h)	1272	1284	1964	504	2316	304	2260	276
R $\times 10^{12}$ (kg/m^{4}s)	3.42	3.52	2.16	8.71	1.96	15.7	0.07	0.55

Table S2. Calculated hydraulic resistances of each device when two PBS buffers were used as a sample and running buffer. μ, w, L, and h stand for the viscosity of fluid, the width, length, and the height of microfluidic channel, respectively.

Device	DLD 1		DLD 2		DLD 3		IT	
	Outlet 1	Outlet 2	Outlet 1	Outlet 2	Outlet 1	Outlet 2	Dummy	Lysis
μ (cP)	1.1	1.1	1.1	1.1	1.1	1.1	1.1	1.1
w (μm)	842	842	1304	347	1434	194	See table 5	
L (μm)	4458	4458	4458	4458	4458	4458	31.5	31.5
h (μm)	26.2	26.2	26.2	26.2	26.2	26.2	90.3	90.18
R $\times 10^{12}$ (kg/m^{4}s)	3.6	3.6	2.3	9	2.1	16.7	0.06	0.49

Table S3. Measured hydraulic resistances of each device when whole blood and PBS were used as a sample and running buffer.

Device	DLD 1		DLD 2		DLD 3		IT	
	Outlet 1	Outlet 2	Outlet 1	Outlet 2	Outlet 1	Outlet 2	Dummy	Lysis
ΔP (Pa)	1794	1786.2	1998.4	1810.6	1921.8	1981.5	66.4	51.3
Q (μl/h)	468	1832	1564	792	1960	416	1992	348
R $\times 10^{12}$ (kg/m^{4}s)	13.8	3.51	4.6	8.23	3.53	15.47	0.12	0.53

Table S4. Calculated hydraulic resistances of each device when whole blood and PBS were used as a sample and running buffer.

Device	DLD 1		DLD 2		DLD 3		IT	
	Outlet 1	Outlet 2	Outlet 1	Outlet 2	Outlet 1	Outlet 2	Dummy	Lysis
μ (cP)	4.1	1.1	2.18	1.1	1.94	1.1	1.94	1.1
w (μm)	842	842	1304	347	1434	194	See table 5	
L (μm)	4458	4458	4458	4458	4458	4458	31.5	31.5
h (μm)	26.2	26.2	26.2	26.2	26.2	26.2	90.3	90.18
R $\times 10^{12}$ (kg/m^{4}s)	14.7	3.6	5.03	9	3.75	16.7	0.12	0.49

Table S5. The channel widths of the dummy and the lysis channel used for hydraulic resistance calculation (Fig S6).

Device	Dummy channel	Lysis channel
w 1 (µm)	222.5	13.2
w 2 (µm)	174.6	11.2
w 3 (µm)	133.6	9.2
w 4 (µm)	101.6	7.2
w 5 (µm)	78.5	5.2
w 6 (µm)	63.8	3.2

Table S6. Comparison of calculated and measured CF values for different DLD devices.

	Measured WBC number (/ml)			Measured outlet volume (µl)			Calculated R (kg/m^4s)		
Device	$\#_{1, blood}$	$\#_{2,DLD}$	$\#_{1, blood}$ / $\#_{2,DLD}$	$V_{1, blood}$	$V_{2,DLD}$	$V_{1, blood}$ / $V_{2,DLD}$	$R_{1, blood}$	$R_{2,DLD}$	$R_{2,DLD}$ / $R_{1, blood}$
DLD 1	4.55×10^6	1.2×10^6	0.26	500	1832	0.27	14.4×10^{12}	3.6×10^{12}	0.25
DLD 2	4.55×10^6	2.7×10^6	0.59	500	792	0.63	14.4×10^{12}	9×10^{12}	0.62
DLD 3	4.55×10^6	5.2×10^6	1.14	500	416	1.12	14.4×10^{12}	16.9×10^{12}	1.17

Table S7. Numerical values and constants used for calculating shear-rate and viscosity values in the microfluidic channel.

Q_{blood} (µl/h)	Q_{PBS} (µl/h)	w_{Blood} (µm)	w_{PBS} (µm)	h (µm)	Shear rate (s^{-1})	Viscosity (cP)
10	40	1550.1	469.8	26.2	15.7	14.3
50	200	1326.1	693.8	26.2	91.5	8.3
100	400	1132.9	887.0	26.2	214.3	5.5
200	800	1071.4	948.5	26.2	453.2	4.9
250	1000	1018.7	1001.2	26.2	595.8	4.4
500	2000	1001.2	1018.7	26.2	1212.5	4.2
